# Urine Caffeine Concentration in Doping Control Samples from 2004 to 2015

**DOI:** 10.3390/nu11020286

**Published:** 2019-01-29

**Authors:** Millán Aguilar-Navarro, Gloria Muñoz, Juan José Salinero, Jesús Muñoz-Guerra, María Fernández-Álvarez, María del Mar Plata, Juan Del Coso

**Affiliations:** 1 Exercise Physiology Laboratory, Camilo José Cela University, 28692 Madrid, Spain; millan.aguilar@ufv.es (M.A.-N.); jjsalinero@ucjc.edu (J.J.S.); 2 Faculty of Education, Francisco de Vitoria University, 28223 Madrid, Spain; 3 Doping Control Laboratory, Spanish Agency for Health Protection in Sport, 28040 Madrid, Spain; gloria.munoz@aepsad.god.es (G.M.); maria.fernandez@aepsad.god.es (M.F.-Á.); 4 Department for Doping Control, Spanish Agency for Health Protection in Sport, 28016 Madrid, Spain; jesus.munoz@aepsad.god.es (J.M.-G.); maria.plata@aepsad.god.es (M.d.M.P.)

**Keywords:** pharmacokinetics, energy drink, exercise, elite athlete, performance

## Abstract

The ergogenic effect of caffeine is well-established, but the extent of its consumption in sport is unknown at the present. The use of caffeine was considered “prohibited” until 2004, but this stimulant was moved from the List of Prohibited Substances to the Monitoring Program of the World Anti-Doping Agency to control its use by monitoring urinary caffeine concentration after competition. However, there is no updated information about the change in the use of caffeine as the result of its inclusion in the Monitoring Program. The aim of this study was to describe the changes in urine caffeine concentration from 2004 to 2015. A total of 7488 urine samples obtained in official competitions held in Spain and corresponding to athletes competing in Olympic sports (2788 in 2004, 2543 in 2008, and 2157 in 2015) were analyzed for urine caffeine concentration. The percentage of samples with detectable caffeine (i.e., >0.1 μg/mL) increased from ~70.1%, in 2004–2008 to 75.7% in 2015. The median urine caffeine concentration in 2015 (0.85 μg/mL) was higher when compared to the median value obtained in 2004 (0.70 μg/mL; *p* < 0.05) and in 2008 (0.70 μg/mL; *p* < 0.05). The urine caffeine concentration significantly increased from 2004 to 2015 in aquatics, athletics, boxing, judo, football, weightlifting, and rowing (*p* < 0.05). However, the sports with the highest urine caffeine concentration in 2015 were cycling, athletics, and rowing. In summary, the concentration of caffeine in the urine samples obtained after competition in Olympic sports in Spain increased from 2004 to 2015, particularly in some disciplines. These data indicate that the use of caffeine has slightly increased since its removal from the list of banned substances, but urine caffeine concentrations suggest that the use of caffeine is moderate in most sport specialties. Athletes of individual sports or athletes of sports with an aerobic-like nature are more prone to using caffeine in competition.

## 1. Introduction

Caffeine (1,3,7-trimethylxanthine) is a stimulant naturally present in a variety of foods and drinks, although it is also artificially included in dietary and sports supplements, over-the-counter medications, and beverages. In the sport setting, caffeine is widely utilized because it might have the capacity to enhance endurance performance [1,2], anaerobic-based performance [3], and strength/power-oriented performance [4,5] in exercise and sports of different nature [6,7,8]. There is strong evidence supporting that caffeine, when ingested prior to exercise, and at a dosage of 3–6 mg per kg of body mass, could benefit sports performance as it has been recently recognized by the International Olympic Committee in its consensus statement on dietary supplements [9]. However, the ergogenicity of caffeine might be affected by the scenario of use and may vary widely among individuals because of several factors that include genetic variants, the microbiome and habituation to caffeine [10]. Specifically, it has been recently found that AA homozygotes for a single nucleotide polymorphism in the CYP1A2 gene (rs762551, also known as −163C>A) might obtain greater ergogenic benefits from acute caffeine intake (2–6 mg/kg) than C-allele carriers [11,12,13], although this is not always the case [14,15,16,17]. In addition, previous investigations have suggested that the ergogenic effect of acute caffeine ingestion (3–5 mg/kg) might be reduced by habitual caffeine intake [18,19], suggesting a progressive tolerance to the ergogenic effects of this substance when this substance is ingested chronically. However, other investigations have shown that naïve/low caffeine consumers benefited from the acute intake of 3–6 mg/kg of caffeine to a similar extent to habitual caffeine consumers [20,21], and, to date, there is not a clear consensus about time course of tolerance to the performance benefits of caffeine. Although the reasons to explain tolerance to caffeine require further investigation, it seems clear that the physiological responses to acute caffeine intake have a great inter-individual variability [22]. 

The use of caffeine in sports can also have several drawbacks, such as increased ratings of nervousness and insomnia [23] that might limit its efficacy to enhance performance. In this respect, the “more is better” philosophy (i.e., >9 mg/kg), when applied to caffeine, may result in a higher prevalence of side effects [24,25] that outweigh the potential performance benefits of this stimulant. Likely due to these and other drawbacks, caffeine was considered a banned substance in sport by the medical commission of the International Olympic Committee and other anti-doping authorities between 1984 and 2004, and its use was prohibited only in competition. A 12 μg/mL threshold for urine caffeine concentration was set in 1987 to limit the use of high doses of caffeine and athletes that surpassed this threshold were penalized for doping misconduct. The World Anti-Doping Agency (WADA) decided to remove caffeine from the list of banned substances with effect from January 1, 2004, and since then, athletes have been able to consume caffeine-containing products freely. However, WADA included caffeine in its Monitoring Program; a program designed to monitor and detect patterns of misuse in substances not included in the prohibited list, but with the possibility of being harmful in sport [26]. Since 2004, WADA has monitored the proportion of urine samples with a caffeine concentration of over 6 μg/mL in order to monitor the use of high doses that could be harmful for athletes, although the data are not public. Interestingly, the concentration of caffeine in the urine samples used for doping control remained similar between 1993–2002 (i.e., when caffeine was in the list of banned substances) [27] and 2004–2008 (i.e., when caffeine was removed from the list of banned substances) [28,29]. These data suggest that the use of caffeine was not substantially modified with the removal of caffeine from the list of banned substances, likely because the “12-μg/mL-threshold” was not an effective deterrent to prevent the use of caffeine to increase physical performance. However, since 2008, there is no investigation that have studied the trends in the use of caffeine sports despite the evidence that support the ergogenicity of caffeine has greatly increased in the last years [1,2,5,30,31]. Thus, the aim of this study was to describe the changes in urine caffeine concentrations in Olympic sports using samples obtained in 2004, 2008, and 2015. The ultimate goal of this study was to use the evolution in urinary caffeine concentration to infer changes in the use of caffeine in sport. 

## 2. Materials and Methods 

For this study, we measured the urine caffeine concentration in all samples submitted to the Madrid Doping Control Laboratory (Spain) in 2004, 2008, and 2015 as part of the WADA Monitoring Program. The samples measured corresponded to specimens gathered after national and international competitions held in Spain, since urine specimens collected out-of-competition are not routinely analyzed for caffeine detection. The current study presents an analysis of the 7488 urine samples that corresponded to athletes competing in Olympic sports (2788 in 2004, 2543 in 2008, and 2157 in 2015). In 2004, 25.4% of the samples pertained to women athletes, 26.0% in 2008 and 24.2% in 2015. To obtain representative data on each sport discipline, a threshold of >25 samples per year was established to include any Olympic sport in the analysis. Information about the athlete’s sex (included on the anti-doping form) was integrated into a database for the analysis. The investigation used anonymized data obtained for the doping control and thus did not require ethical approval. Participants’ rights and confidentiality were protected during the whole study, and the data were only used for the purposes included in this investigation. The study conformed to the Declaration of Helsinki. 

### 2.1. Urine Analysis

All samples were obtained following the Guidelines for Urine Sample Collection described by WADA [32]. Upon collection, the samples were sent to the Doping Control Laboratory by special refrigerated transport and arrived at the laboratory with an anonymized format (alpha-numeric code). After arrival, a portion of the sample was used to measure urine caffeine concentration and the remaining amount was destined to other anti-doping purposes. Specifically, a portion (5 mL) of each urine sample was poured into a 15-mL screw-capped glass tube. Then, 50 µL of internal standard (diphenylamine 100 µg/mL) was added to the sample. After that, 100 µL of sodium hydroxide 10 mol/L and 0.5 g of sodium sulphate were added to increase the transfer of analytes from the aqueous to the organic phase. Alkaline extraction was performed by adding 5 mL of methyl tert-butyl ether and centrifuging the sample at 60 rpm for 20 min. After that, the sample was frozen in a cryogenic bath, and the organic phase (upper phase and not frozen) was transferred to a clean vial. The extract was concentrated with nitrogen, and 2 µL of the remaining extract was injected into the system for caffeine quantification. 

The methodology to quantify urine caffeine concentration was based on gas chromatography–mass spectrometry (GC-MS), and was validated according to ISO17025. The measurement of each batch of urine samples was preceded by a calibration process, using a solution with an established caffeine concentration (6 μg/mL). GC-MS analysis was performed using a 6890N Gas Chromatograph (Agilent Technologies, Santa Clara, CA, USA) coupled to a 5973N Mass Selective Detector (Agilent Technologies). All the chromatograms in the samples analyzed in 2004 and in 2008 were obtained in the scan mode range. At this time, the GC was equipped with a fused silica capillary column OV-1 (J & W Scientific Inc., Folsom, CA, USA). In 2015, the chromatograms were obtained in the single ion monitoring (SIM) mode and the GC was equipped with a capillary column Ultra-1 (J & W Scientific Inc., Folsom, CA, USA). In all analyses the carrier gas was helium, and they were carried out at a constant pressure of 15 psi. To facilitate separation, the initial column temperature was set at 90 °C and the final column temperature was set at 300 °C. The temperature on the injector port was set at 275 °C.

### 2.2. Validation Procedure

The between-days reproducibility was evaluated using 20 measurements of the calibration solution obtained over two months. The between-days coefficient of variation (at 6 μg/mL) was 7%. Accuracy was calculated in terms of the recovery factor (experimental value/theoretical value, expressed as a percentage). The value obtained was 105%, and no tendencies were observed. Combined uncertainty was estimated taking into account the contributions of accuracy and reproducibility and the value obtained was 11%. The limit of detection (LOD) was 0.1 μg/mL.

### 2.3. Statistical Analysis

All samples with a urinary caffeine concentration below the LOD were considered to be specimens without any caffeine content. The remaining samples were categorized into intervals of 1.0 μg/mL, with a maximal caffeine concentration of 13.0 μg/mL. Most of the samples had a urinary caffeine concentration between 0.0 and 13.0 μg/mL, but 32 samples had a urinary caffeine concentration of >13.0 μg/mL (14 in 2004, 11 in 2008, and 7 in 2015). These samples were included in the statistical analysis, but they were not included in the graphical presentation of the data per 1.0 μg/mL-categories. The samples were grouped by sport discipline, by year of collection, and by athlete’s sex. Normality for each year of collection was tested with the Kolmogorov-Smirnov test. 

Data are presented as median ± and interquartile range (25% and 75% percentile) for quantitative variables (urine caffeine concentration), while qualitative variables (distribution) are presented as percentages. Urine caffeine concentration had a non-normal distribution and thus, non-parametric statistics were later employed. The comparison of the urine caffeine concentration among the three years (2004 vs. 2008 vs. 2015) was tested with the Kruskal-Wallis test. The changes in the evolution of the urine caffeine concentration within each sport were also identified with the Kruskal-Wallis test. The differences in distribution of samples among ranges of urine caffeine concentration were tested with crosstab and Chi Square tests, including adjusted standardized residuals. The comparison among sport specialties was only performed for the samples obtained in 2015 because a previous publication provided this comparison for 2004–2008 [29]. Finally, the differences between sexes were analyzed with the *U*-Mann Whitney test. The data were analyzed with the statistical package SPSS v 21.0 (SPSS Inc., Chicago, IL, USA). The significance level for all these statistical analyses was set at *p* < 0.05.

## 3. Results

**Figure 1:**
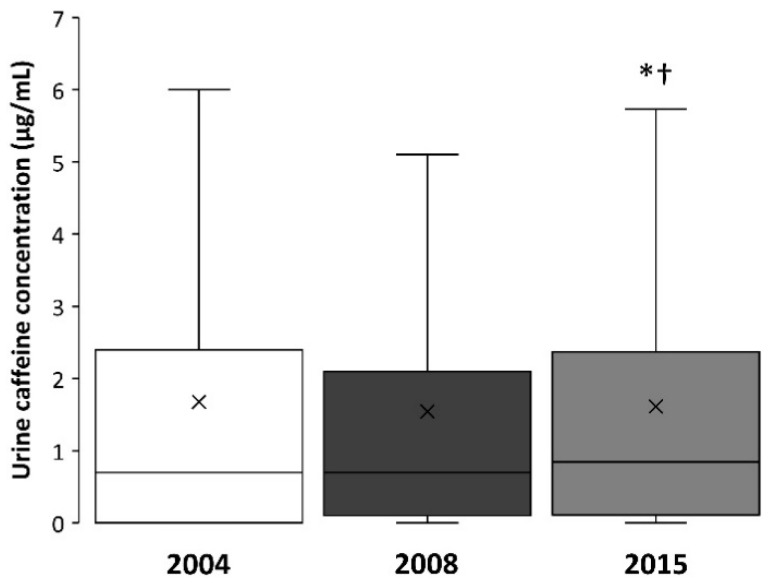
Box-and-whisker plot for caffeine concentration in the urine samples of Olympic sports collected in 2004, 2008, and 2015. The cross depicts the mean value while the lower, middle and upper lines of the box represent the 25%, 50% and 75% percentile. Whiskers represent 1.5 × interquartile range. Outlier data have been removed to facilitate the comprehension of the figure. (*) Different from 2004 at *p* < 0.05; (†) Different from 2008 at *p* < 0.05.

The median urine caffeine concentration in 2015 (0.9; 0.1–2.4 μg/mL) was higher when compared to the median value obtained in 2004 (0.7; 0.0–2.4 μg/mL; *p* < 0.05) and 2008 (0.70; 0.1–2.1 μg/mL; *p* < 0.05; Figure 1). The maximal value of caffeine concentration was 21.1, 19.2 and 18.6 μg/mL for 2004, 2008, and 2015, respectively.

**Figure 2:**
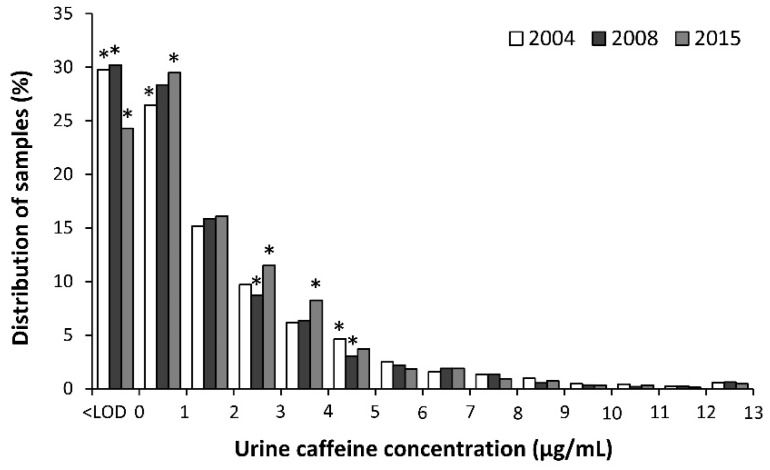
Distribution of urine samples according to the concentration of caffeine in 2004, 2008, and 2015. (*) Different from the expected value. LOD: limit of detection.

Figure 2 depicts the distribution of urine samples in each year of analysis according to their urine caffeine concentration, using 1 μg/mL intervals. The distribution of the samples was slightly different among these years because in 2015, the percentage of samples below the limit of detection was lower than expected (*p* < 0.05) while the percentage of samples between 2 and 4 μg/mL was higher than expected (*p* < 0.05). The percentage of samples with detectable caffeine (i.e., > 0.1 μg/mL) was 70.3%, 69.8%, and 75.7% in 2004, 2008, and 2015, respectively. The proportion of samples with urine caffeine concentrations of >12 μg/mL was 0.79%, 0.87%, and 0.60% in 2004, 2008, and 2015, respectively.

**Figure 3:**
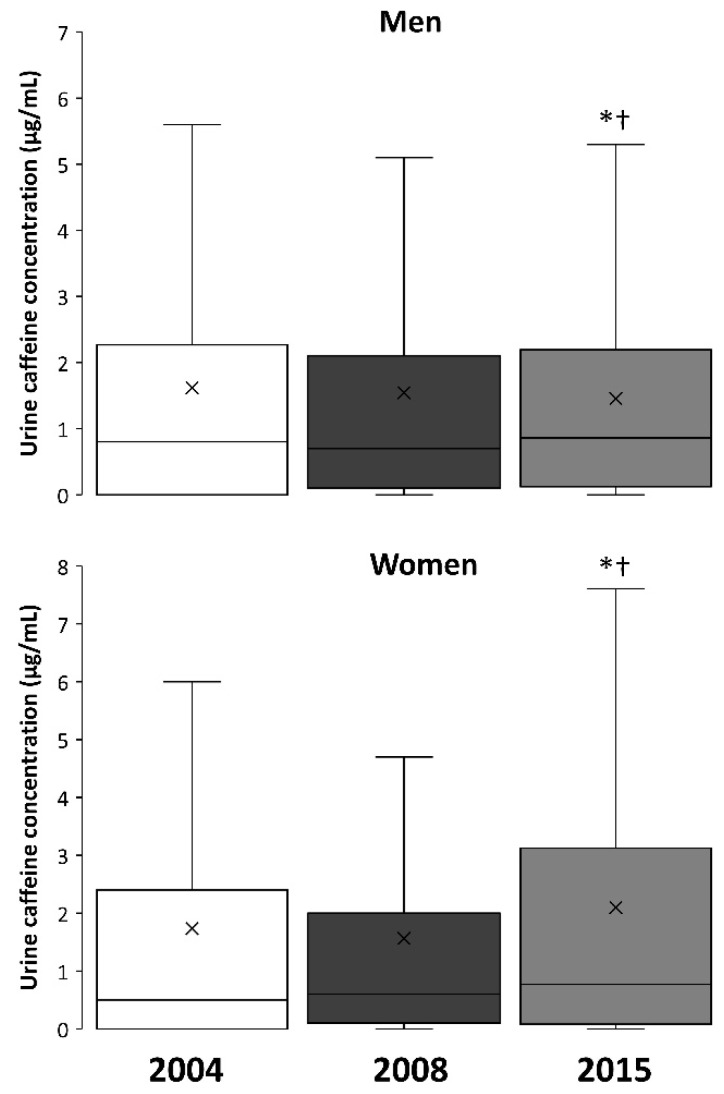
Box-and-whisker plot for caffeine concentrations in the urine samples from men and women collected in 2004, 2008, and 2015. The cross depicts the mean value while the lower, middle and upper lines of the box represent the 25%, 50%, and 75% percentile. Whiskers represent 1.5 × interquartile range. Outlier data have been removed to facilitate the comprehension of the figure. (*) Different from 2004 at *p* < 0.05; (†) Different from 2008 at *p* < 0.05.

Figure 3 depicts box-and-whisker plots for the changes in urine caffeine concentrations in 2004, 2008, and 2015 in men and women. The median values obtained in 2015 were different from 2004 and 2008 in men (upper panel) and women (lower panel), respectively (*p* < 0.05), while the median values were always higher in men than in women (*p* < 0.05). 

**Figure 4:**
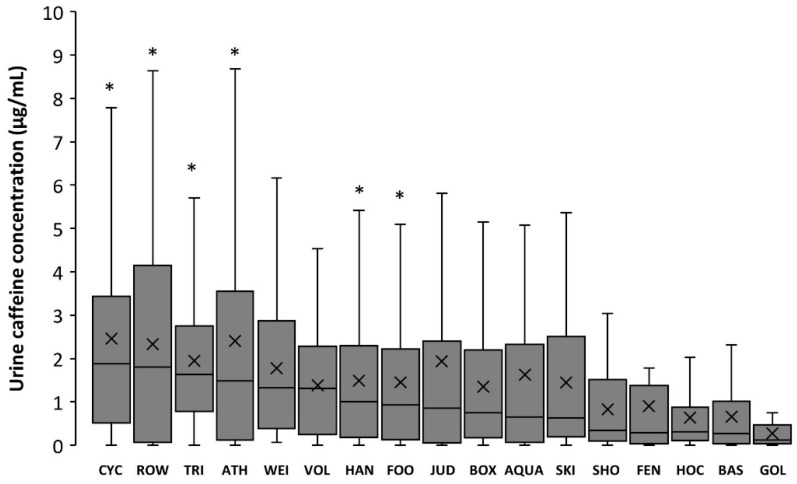
Box-and-whisker plot for caffeine concentrations in the urine samples of Olympic sports collected in 2015. The cross depicts the mean value while the lower, middle, and upper lines of the box represent the 25%, 50%, and 75% percentile. Whiskers represent 1.5 × interquartile range. Outlier data have been removed to facilitate the comprehension of the figure. CYC = Cycling; ROW = Rowing; TRI = Triathlon; ATH = Athletics; WEI = Weightlifting; VOL = Volleyball; HAN = Handball; FOO = Football; JUD = Judo; BOX = Boxing; AQUA = Aquatics; SKI = Skiing; SHO = Shooting; FEN = Fencing; HOC = Hockey; BAS = Basketball; GOL = Golf. (*) Different from GOL at *p* < 0.05.

Figure 4 depicts urine caffeine concentration in Olympic sports in 2015 using box-and-whisker plots. The sports with the highest concentration of caffeine in urine were cycling, rowing, triathlon, athletics, weightlifting, and volleyball (all with median values >1.0 μg/mL); the sports with the lowest urine caffeine concentration were shooting, fencing, hockey, basketball, and golf (all with median values <0.5 μg/mL). Golf presented urine caffeine concentrations lower than cycling, athletics, rowing, triathlon, handball, and football (*p* < 0.05). Table 1 contains information on the changes in the median urine caffeine concentrations in Olympics sports for the years 2004, 2008, and 2015. Specifically, the values obtained in 2015 were significantly higher than those obtained in 2004 and 2008 in aquatics, athletics, boxing, judo, and football. In golf and skiing, the data from 2015 were higher only when compared to 2008, while in rowing and weightlifting, the values in 2015 were only different to 2004. 

## 4. Discussion

The purpose of this investigation was to describe the changes in urine caffeine concentration of samples obtained in competition of Olympic sports for the years 2004, 2008, and 2015. The final goal was to determine the evolution in the use of caffeine in sports, especially one decade after it was removed from the banned list. For this purpose, we measured caffeine concentration in 7488 urine samples received by the WADA-accredited Doping Control Laboratory in Madrid as part of the Monitoring Program. The main outcomes of this investigation indicate the following: (a) in 2015, there was a slight but statistically significant increase in urine caffeine concentration when compared to both 2004 and 2008. This increase is reflected by a lower proportion of athletes with urinary caffeine concentrations below the limit of detection and a higher proportion of athletes with concentrations between 2 and 4 μg/mL; (b) the increase in urine caffeine concentration in 2015 was similarly present in both men and women but it was unequal in all sport disciplines. Sports such as aquatics, athletics, boxing, judo and weightlifting had a progressive increase in urine caffeine concentration from 2004 to 2015, while the concentration in other Olympic sports remained stable throughout this period; (c) in 2015, cycling, athletics, and rowing were the sports with the highest urine caffeine concentration, while shooting, basketball, and golf were the disciplines with the lowest concentrations of urinary caffeine. All this information suggests that the use of caffeine in sports increased from 2008 to 2015, particularly in some individual sports. However, the magnitude of the change in the urine caffeine concentrations obtained in competition does not reflect misuse of this substance in most sport disciplines.

After the removal of caffeine from the list of prohibited substances in 2004, athletes were free to consume caffeine at any amount before, during or even after competitions without the burden of being sanctioned by the anti-doping authorities. In the first five years after this administrative decision, the urinary concentration of caffeine in sport did not significantly change, as was shown by the comparative values of the reports made before [27] and after 2004 [28,29]. The absence of change suggested a high but stable utilization of caffeine by athletes, with most of the samples in the low-to-middle range of urinary caffeine concentrations. However, more than 300 new studies dealing with the effects of caffeine in sports have appeared since 2008, particularly original investigations determining the effects of caffeine on team sports, strength- and power-based sports or those with an intermittent nature. Besides, caffeine-containing products have become more accessible in all types of markets because of the conception of new supplements that incorporate caffeine in their formulation (e.g., pre-work-outs, carbohydrate gels, etc.) or the increase in the popularity of caffeinated drinks. Even so, the use of caffeine in sports competition has not dramatically changed since 2008 although a slight increase in 2015 is suggested by the changes in the distribution of urine caffeine concentration. First, the percentage of samples with a urine caffeine concentration below the limit of detection decreased from 31.2 in 2008 to 24.3% in 2015 (Figure 2), indicating that the proportion of athletes that do not consume caffeine before or during sports competition has slightly shrunk in the last few years. Furthermore, the proportion of athletes with urine caffeine concentrations in the range of 2–4 µg/mL increased in 2015. Thus, it can be suggested that caffeine is a recurrent substance used by ~75% of athletes in competition with a minor but significant evolution towards a higher use in sports in 2015.

Caffeine is a substance present in a multitude of foods and drinks, but the amount of caffeine included in most commercially available products with caffeine has not been shown to have a clear effect on physical performance (a dose of at least 3 mg/kg is usually necessary to increase performance [4,9]). The omnipresence of caffeine in the diet means that this substance can be consumed by some athletes without the intention of increasing physical performance (i.e., social use of caffeine). Although there is no consensus about the urinary caffeine concentration that differentiates the social use of caffeine from the intentional use of caffeine to enhance performance, previous investigations have revealed that lower doses of caffeine that increase performance (i.e., 3–6 mg/kg of body mass) derive in urinary caffeine concentrations of 2–5 µg/mL after simulated and real competitions [33,34,35] or other forms of exercise [36]. Despite this evidence, WADA only considers relevant, in terms of misuse and abuse of caffeine, those samples with urinary caffeine concentration of above 6 µg/mL [32] despite the fact that this might be indicative of caffeine dosages of >9 mg/kg [37]. In the current data, the proportion of samples above 6 µg of caffeine per mL of urine was 5.9%, 5.4%, and 4.8% for 2004, 2008, and 2015, respectively. By using the cut-off point proposed by WADA, one might assume that caffeine abuse has remained constant and low in the last decade. However, urinary caffeine concentrations between 2 and 6 µg/mL might also be indicative of intentional use of caffeine in sports. 

Interestingly, the increase in the concentration of caffeine has not been equally present in all sports. The mean urinary concentration of athletes tested in aquatics, athletics, boxing, judo, and weightlifting increased from 2004 to 2015, suggesting a rise in the use of this substance among these particular sports. Other sports such as basketball, cycling, fencing, handball, hockey, shooting, and volleyball have maintained urine caffeine concentration at relatively stable values, suggestive of a steady-state use of caffeine in the last decade. Despite the uneven evolution or urinary caffeine concentration from 2004 to 2015 among sports, the individual disciplines with an aerobic-based performance continue to be the sports with the highest concentrations of caffeine, while team sports and accuracy sports are the disciplines with the lowest concentrations of caffeine (Figure 4). The higher urinary caffeine concentrations found in aerobic-based sports might be related to the traditional evidence that supported the ergogenic effects of caffeine by using laboratory-based research protocols with endurance-like exercise. However, more recent evidences, obtained in sport-specific situations, have demonstrated that the beneficial effects of pre-competition caffeine intake is extended to sprint- and power-based exercise [5,38], team sports [6,39,40], combat sports [8,41] and sports in which accuracy is a key element for success [42,43]. With these new evidences, it might be expected a higher consumption of caffeine—and a higher urinary caffeine concentration—in these type of sport disciplines in the next years that should be investigated in future research. 

The urinary concentration of caffeine has significantly increased in both male and female athletes since 2004 (Figure 2) and median values reached 0.9 (0.1–2.2) and 0.8 (0.1–3.1) μg/mL, respectively, in 2015. Although the median values for men and women are very comparable, the proportion of samples from women athletes at high urinary caffeine concentrations is higher than expected in comparison to the proportion of urine samples from male athletes. For example, ~65.0% of all urine samples with a concentration >10 μg/mL corresponded to female participants, despite urine samples from women representing only about 25.3% of all the samples analyzed. In the opinion of these authors, the higher incidence of women’s samples in the highest ranges of urinary concentrations of caffeine could be the result of the unintended intake of larger relative doses of caffeine, in terms of mg per kg of body mass. Caffeine-containing products are equally available in the market for both men and women, but the habitual lower mean body mass of female athletes might mean that the same absolute amount of caffeine ingested (for example, 160 mg of caffeine in a 500 mL can of an energy drink) results in a higher relative dose in mg/kg. This is also supported by the similar urinary pharmacokinetic parameters found for male and female adults [44], which suggests that the higher urinary caffeine excretion in women is related to the ingestion of higher relative doses rather than differences in caffeine metabolism and excretion. 

The current analysis presents some limitations that should be discussed to correctly understand the outcomes of the investigation. First, the analysis included data from urine caffeine concentration in three selected years (2004, 2008, and 2015). According to WADA’s Monitoring Program specifications, only urine samples with a urinary caffeine concentration above 6.0 μg/mL had to be reported to WADA (and those samples with concentrations below this cut-off remained unreported. Thus, due to the high number of samples analyzed in the Madrid Doping Control Laboratory between 2004 and 2015, we have been only able of obtaining the data of all urine samples, irrespective of their urinary caffeine concentration, in these three specific years. Second, the urine samples included in the analysis were exclusively obtained in national and international competitions held in Spain. Although in these competitions participate athletes of different nationalities, it is expected that a high proportion of the samples analyzed pertained to Spanish athletes. Thus, it is still possible that the evolution of urinary caffeine concentration could have been different in other countries due to social, genetic and lifestyle factors. In addition, the absence of out-of-competition urine samples impeded us to have a control to differentiate the use of caffeine on a day-to-day basis vs. the use before sports competition. Third, absorption, distribution, metabolism, and excretion of caffeine in the human body is affected by a myriad of genetic and environmental factors [45] that could affect the concentration of caffeine in urine in individuals taking the same dose before exercise. Post-competition urinary caffeine levels might be affected by the timing of the urine sample in relation to the caffeine dose [46] or the opportunities to urinate during or after an event. In this regard, the sport disciplines analyzed in this investigation have different regulations, particularly different durations or the presence of several competitions within the same day. Since caffeine is typically consumed before exercise, a longer competition period might allow more time for metabolism and excretion of the substance, affecting those sports with longer competition durations. In addition, caffeine could be ingested more than once in long-lasting events to maintain the effects of the substance on performance. Nevertheless, we believe that the high number of samples analyzed per year minimizes the effect of these factors on the outcomes of the investigation, and the authors believe that the data provided by this research reflect the evolution of the use of caffeine in sports. 

## 5. Conclusions

In summary, the concentration of caffeine in the urine samples obtained after competition in Olympic sports increased from 2004 to 2015, which might indicate a slightly higher use of this substance in both men and women athletes. The analysis by disciplines revealed that some, but not all, sports have shown increases in the concentration of urinary caffeine, suggesting that the popularity of this substance has grown in some sports. Athletes of individual sports or athletes of sports with an aerobic-like nature are more prone to using caffeine in competition. Finally, investigations about the effects of caffeine on female athlete populations should be promoted because women athletes present slightly higher urinary concentrations than men counterparts. 

## Figures and Tables

**Table 1:** Urine caffeine concentrations (μg/mL) in Olympic sports in 2004, 2008, and 2015. Data are medians (25% and 75% percentile) for each sport.

Sport	2004	2008	2015	*p* Value
Aquatics	0.1 (0.0–0.8)	0.1 (0.0–1.2)	0.7 (0.1–2.3) *^†^	<0.01
Athletics	0.7 (0.0–2.6)	0.8 (0.1–2.4)	1.5 (0.1–3.6) *^†^	<0.01
Basketball	0.2 (0.0–0.9)	0.4 (0.0–1.2)	0.3 (0.1–1.0)	0.13
Boxing	0.5 (0.0–0.9)	0.0 (0.0–0.8)	0.8 (0.2–2.2) *^†^	<0.01
Cycling	2.0 (0.5–4.0)	1.7 (0.5–3.6)	1.9 (0.5–3.4)	0.30
Fencing	0.5 (0.0–0.9)	0.1 (0.0–0.8)	0.3 (0.1–1.4)	0.19
Football	0.7 (0.0–2.0)	0.5 (0.1–1.6)	0.9 (0.1–2.2) *^†^	<0.01
Golf	0.0 (0.2–0.4)	0.0 (0.0–0.0) *	0.1 (0.0–0.5) ^†^	<0.01
Handball	1.0 (0.2–2.7)	0.9 (0.1–2.1)	1.0 (0.2–2.3)	0.40
Hockey	0.4 (0.0–1.6)	0.9 (0.2–2.2)	0.3 (0.3–0.9)	0.60
Judo	0.2 (0.0–0.8)	0.2 (0.0–0.5)	0.9 (0.1–2.4) *^†^	<0.01
Rowing	0.4 (0.1–1.6)	2.7 (0.1–5.0) *	1.8 (0.1–4.1) *	<0.01
Shooting	0.4 (0.0–2.0)	0.1 (0.0–1.7)	0.3 (0.1–1.5)	0.24
Skiing	0.2 (0.0–1.0)	0.3 (0.1–0.9)	0.6 (0.2–2.5) ^†^	0.03
Triathlon	1.2 (0.3–4.2)	3.0 (1.5–6.2) *	1.6 (0.8–2.8)	<0.01
Volleyball	0.9 (0.1–2.0)	1.5 (0.2–2.6)	1.3 (0.3–2.2)	0.45
Weightlifting	0.2 (0.0–1.2)	0.6 (0.0–1.8)	1.3 (0.4–2.9) *^†^	0.01

(*) Different from 2004 at *p* < 0.05. (†) Different from 2008 at *p* < 0.05.
